# Diabetic Striatopathy Complicated With Acute Ischemic Stroke: A Case Report

**DOI:** 10.3389/fnins.2022.877479

**Published:** 2022-07-12

**Authors:** Xiao Huang, Junli Qi, Yiding Li, Jianhui Li, Meng-Ge Yang

**Affiliations:** ^1^Department of Neurology, The Second Affiliated Hospital of Henan University of Science and Technology, Luoyang, China; ^2^Department of Orthopaedic Surgery, The Second Affiliated Hospital, Henan University of Science and Technology, Luoyang, China; ^3^Department of Neurology, Tongji Hospital of Tongji Medical College, Huazhong University of Science and Technology, Wuhan, China

**Keywords:** diabetic striatopathy, acute ischemic stroke, hyperglycemia, involuntary movement, chorea

## Abstract

Diabetic striatopathy (DS) is a rare complication secondary to hyperglycemia, featured by the choreiform movements and reversible striatal abnormalities on neuroimaging. Several studies have described the clinical characteristics of DS, however, the simultaneous occurrence of DS and acute ischemic stroke (AIS) in the striatum has not been reported. Herein, we report a 68-year-old man with uncontrolled type 2 diabetes who experienced the progressive involuntary movement of the right upper and lower limbs for 10 days. We initially considered this patient as an AIS with hemorrhage in the left basal ganglia and adjacent area because his brain magnetic resonance imaging (MRI) showed hyperintensity on fluid-attenuated inversion recovery (FLAIR) and diffusion-weighted imaging (DWI) images, as well as slight T1-hyperintensity around T1-hypointensity. However, his symptoms worsen persistently, which was inconsistent with neuroimaging findings. Further computed tomography (CT) scan revealed an extensive hyper-density and focal low-density in the left striatum, suggesting the diagnosis of DS and AIS. His symptoms were in complete remission after 2 months of glucose control. However, striatal hyperintensity on T1 images was significantly increased compared to the initial images, which disappeared 18 months later. Additionally, DWI hyperintensity on infarction lesions disappeared, while softening lesions and gliosis were observed on the follow-up MRI images. Therefore, we finally diagnosed the patient as DS complicated with AIS. This report highlights that DS and AIS could occur simultaneously in the striatum after hyperglycemia, which is easily misdiagnosed as AIS with hemorrhage and requires clinicians to pay more attention to avoid misdiagnosis and delayed treatment.

## Introduction

Diabetic striatopathy (DS) is a rare complication secondary to hyperglycemia, featured by the choreiform movements, and reversible striatal abnormalities in neuroimaging (Chua et al., 2020). In recent years, an increasing number of studies have described the clinical characteristics of DS (Kim et al., 2015; Yu et al., 2017; Wang et al., 2020; Zheng et al., 2020; Zhao et al., 2021; Dubey et al., 2022; Tsalta-Mladenov et al., 2022). However, the simultaneous occurrence of DS and acute ischemic stroke (AIS) in the striatum has not been reported. Herein, we described an unusual case who suffered from DS and AIS simultaneously after hyperglycemia. We also recorded neuroimaging manifestations of this patient at different stages. This report will increase our knowledge of the rare complications of hyperglycemia and help to rapid clinical identification and intervention.

## Case Presentation

A 68-year-old Chinese man suffered from progressive involuntary movements of the right upper and lower limbs for 10 days. Initially, his symptoms were mild and only manifested as a tremor of the right limb. In the following 10 days, his symptoms gradually worsened and presented with gross, purposeless, irregular, rapid choreiform movements, affecting daily life (Supplementary Video 1). The movements were incontrollable but stopped during sleep. Physical examination revealed no obvious signs of weakness in his right extremities, but for continuous choreoathetosis and slightly reduced muscle tone.

This patient had type 2 diabetes for 10 years but has not received any hypoglycemic therapy for nearly half a year. His fasting blood glucose was 20.2 mmol/L (normal range: 3.9–6.1 mmol/L), glycosylated hemoglobin was 14.4% (normal range: 4–6.5 mmol/L) and urine glucose was strongly positive (++++). His urine acetone bodies were positive (+) on admission but turned negative after 2 days. Serum cholesterol was 5.78 mmol/L (normal range: 2.83–5.17 mmol/L). Other laboratory test indexes, including blood routine, liver and renal function, thyroid function, ceruloplasmin, serum electrolyte, and autoimmune antibodies, were normal. No Kayser–Fleischer ring was observed in the cornea. Additionally, this patient was not exposed to any drugs or toxic substances. Detailed laboratory findings are shown in Table 1.

**Table 1:** Laboratory findings at the first day of hospital admission.

**Items**	**Results**	**Reference range**
Age (years)	68	-
Gender	Male	-
Medical history	type 2 diabetes	-
Laboratory findings		
White blood cell count, 10∧9/L	5.36	3.5–9.5
Neutrophil count, 10∧9/L	3.74	1.8–6.3
Lymphocyte counts, 10∧9/L	1.34	1.1–3.2
Fasting blood glucose, mmol/L	20.2	3.89–6.11
Hemoglobin A1c, %	14.4	4–6.5
Total cholesterol, mmol/L	5.78	2.83–5.71
LDL, mmol/L	3.6	0–41
ALT, U/L	15	9–50
AST, U/L	20	15–40
Urea nitrogen, mmol/L	5.6	2.5–6.4
Creatinine, μmol/L	47	60–120
Sodium, mmol/L	137.2	137–147
Potassium, mmol/L	4.77	3.5–5.3
Free Triiodothyronine	3.03	3.1–6.8
Free Thyroxine	18.05	12–22
Antinuclear antibody	Negative	Negative
HIV antibody	Negative	Negative
Treponema pallidum antibody	Negative	Negative
Urine glucose	4+	Negative
Urine ketone	1+	Negative

*LDL, low density lipoprotein; ALT, alanine aminotransferase; AST, aspartate aminotransferase; HIV, human immunodeficiency virus*.

**Figure 1:**
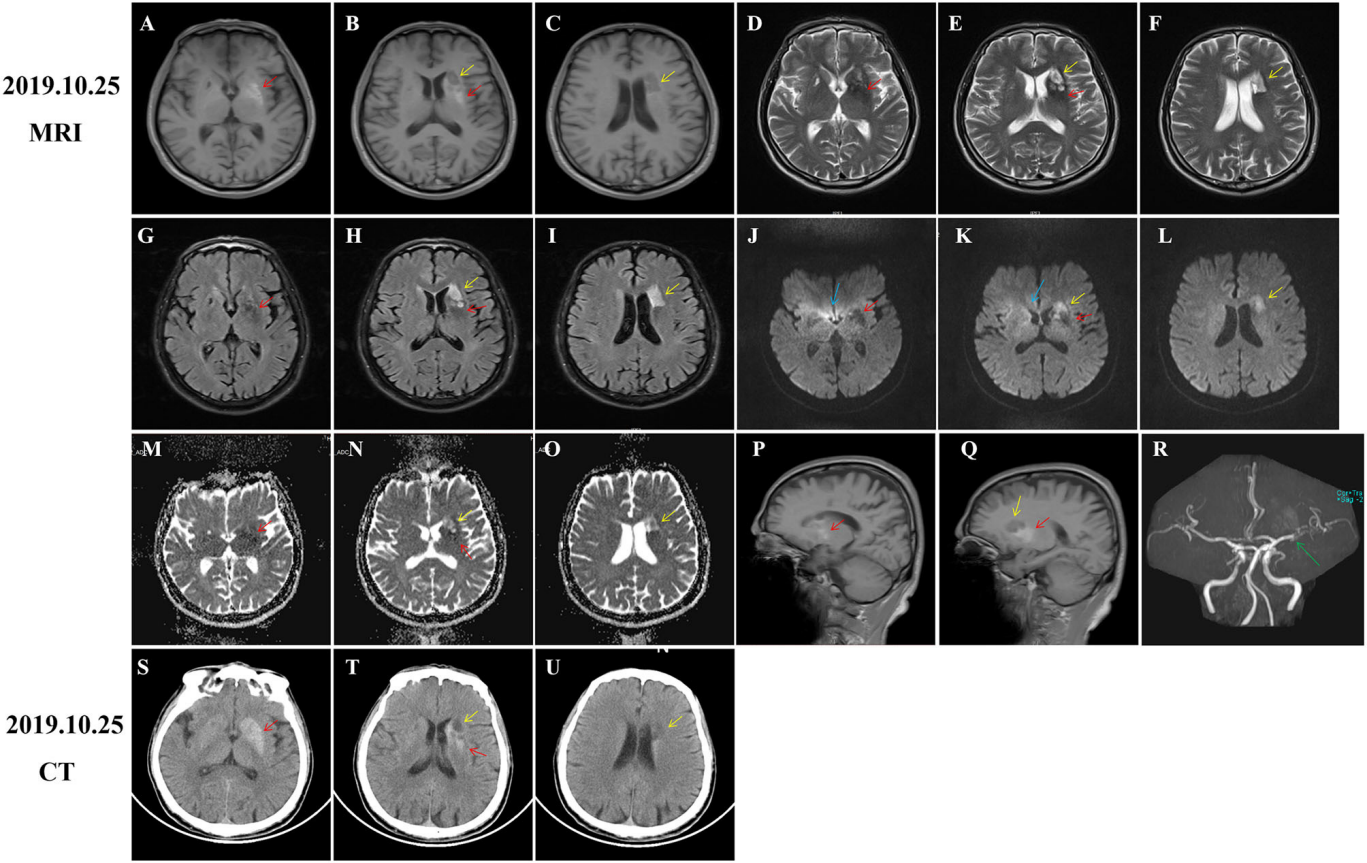
Computed tomography (CT) and MRI of brain at admission. Diabetic striatopathy (DS)-associated striatal abnormalities were marked by red arrows, while infarction focuses were marked by yellow arrows. Typical imaging manifestations of DS were found in the left striatum with T1 hyperintensity [**(A,B)**, coronal images; **(P,Q)**, sagittal images] on MRI and hyper-density on CT images **(S,T)**. The hypointensity on T2 **(D,E)**, fluid-attenuation inversion recovery (FLAIR) **(G,H)**, and apparent diffusion coefficient (ADC) **(M,N)** images, as well as equal signal intensity on DWI images **(J,K)** were also observed in DS-associated striatal lesions. Subacute cerebral infarctions showed T1 hypointensity [**(B,C)** coronal images; **(Q)** sagittal image], T2 hyperintensity **(E,F)**, FLAIR hyperintensity **(H,I)**, DWI hyperintensity **(K,L)**, and slight ADC hyperintensity **(N,O)** on MRI images, as well as hypo-density on CT images **(T,U)**. MRA images disclosed obvious stenosis of the left middle cerebral artery [**(R)**, green arrow]. There were several artifacts on ADC images [**(J,K)**, blue arrows].

His brain MRI showed T1-hypointensity, T2-, and FLAIR- and DWI-hyperintensity in the left striatum and adjacent areas, suggesting acute cerebral infarction lesions (Figures 1B,C,E,F,H,I,K,L, yellow arrows). Besides, slight hyperintensity on apparent diffusion coefficient (ADC) images (Figures 1N,O, yellow arrows) and T1-hyperintensity around T1-hypointensity (Figures 1A,B, red arrows) were also observed. We considered these lesions as subacute infarction lesions with hemorrhage for the first time. Consistently, MRA images also disclosed severe atherosclerosis and stenosis of the left middle cerebral artery (MCA) (Figure 1R, green arrow). However, he presented with progressive involuntary movements, which was inconsistent with neuroimaging findings. Therefore, brain CT was performed and showed an extensive hyper-density (Figures 1S,T, red arrows) and focal low-density (Figures 1T,U, yellow arrows). Combined with the patient's clinical data and neuroimaging findings, we thought that diabetes-related striatum lesions were responsible for clinical attack and progress. The infarction lesions may occur early after onset, or this patient suffered from the subclinical ischemic event before onset. Because ADC images usually showed a low signal within 1 week after a stroke, it gradually normalized and increased to a supra-normal signal (Lansberg et al., 2001; Schulz et al., 2007; Sylaja et al., 2007).

**Figure 2:**
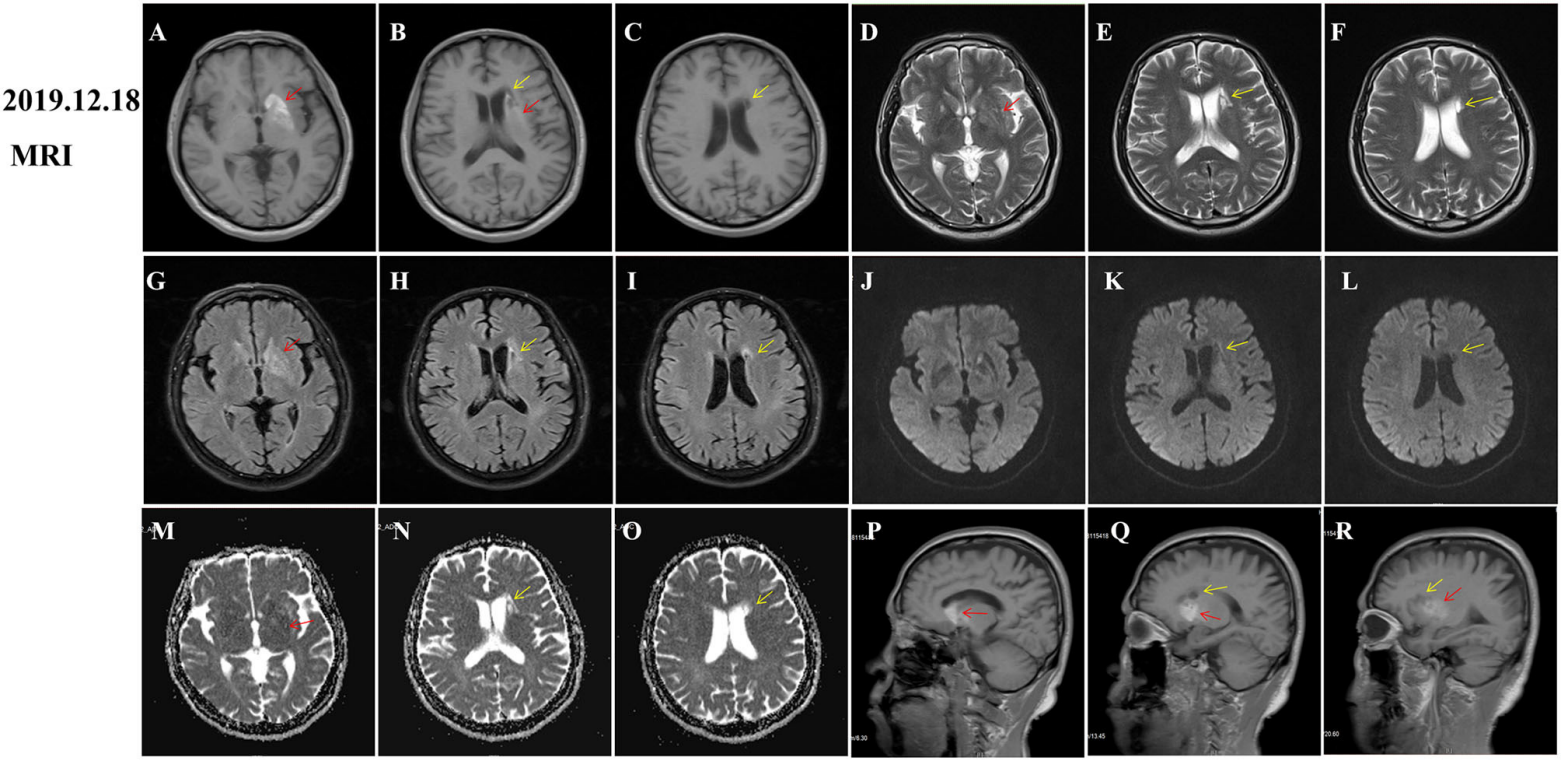
Follow-up magnetic resonance imaging (MRI) of brain 2 months after onset. Diabetic striatopathy (DS)-associated striatal abnormalities were marked by red arrows, while infarction focuses were marked by yellow arrows. DS-associated T1 hyperintensity on the left striatum was obviously extended [**(A)**, coronal image; **(P,Q) (R)**: sagittal images). DS-associated T2 and FLAIR hypointensity changed to slight hyperintensity **(D,G)**. The left striatum showed an equal signal intensity on DWI images **(J)**. DS-associated initial ADC hypointensity changed to equal signal intensity **(M)**. DWI hyperintensity on infarction lesions disappeared **(K,L)** and ADC hyperintensity increased **(N,O)**. Abnormal T1 hypointensity **(B,C)**, T2 hyperintensity **(E,F)**, and FLAIR hypointensity on the infarction core **(H,I)**, as well as FLAIR hyperintensity around the infarction core **(H,I)** suggested the formation of softening lesions and secondary gliosis.

**Figure 3:**
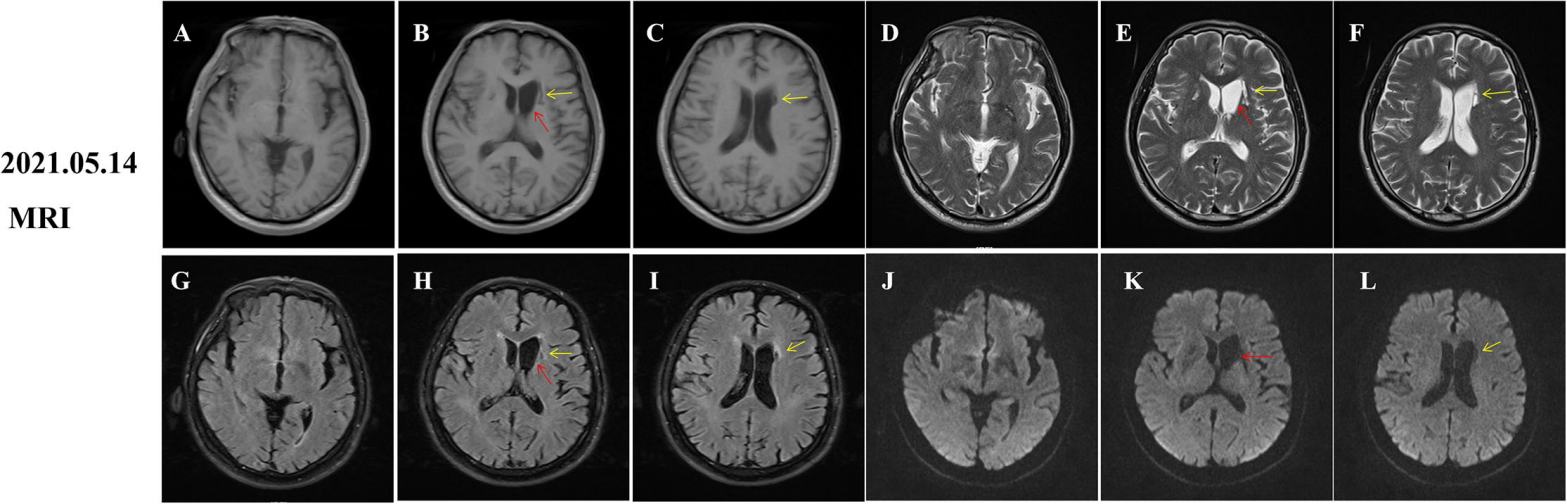
Follow-up magnetic resonance imaging (MRI) of brain 18 months after onset. Diabetic striatopathy (DS)-associated striatal hyperintensity on T1 and T2 and fluid-attenuation inversion recovery (FLAIR) images were disappeared **(A,D,G,J)**, but atrophy of the left caudate nucleus with resultant dilatation of frontal horn of left lateral ventricle was noted [**(B,E,H,K)** red arrows]. Remote cerebral infarctions were observed on T1, T2, FLAIR and diffusion weighted imaging (DWI) images [**(B,C,E,F,H,I,K,L)**, yellow arrows].

During hospitalization, insulin and statins were administrated to control the blood glucose and hyperlipemia, respectively. Aspirin was not given until discharge from the hospital considering the potential risk of bleeding. The involuntary movements were not improved and even worsen after haloperidol treatment. Then, he was initiated on trihexyphenidyl and clonazepam after haloperidol was discontinued. His symptom was significantly improved after 1 week and completely relieved at the 2-month follow-up (Supplementary video 2). However, hyperintensity on T1-weighted MRI images within the striatum was increased compared to that of the initial images (Figures 2A,P–R, red arrows). Given the improvement of symptoms and potential drug side effects, trihexyphenidyl and clonazepam were gradually withdrawn. During the next 18 months of follow-up, the patient's blood glucose was well-controlled and involuntary movements did not relapse. Moreover, a re-examination of brain MRI showed that the striatal hyperintensity on T1 images has disappeared (Figure 3A) and atrophy of the left caudate nucleus with resultant dilatation of the frontal horn of the left lateral ventricle was noted (Figures 3B,E,H,K, red arrows). Additionally, DWI hyperintensity on infarction lesions disappeared on the follow-up MRI (Figures 2K,L,3K,L) while ADC hyperintensity increased (Figures 2N,O). Abnormal T1 hypointensity, T2 hyperintensity, and FLAIR hypointensity on the infarction core, as well as FLAIR hyperintensity around the infarction core, suggested the formation of softening lesions and secondary gliosis (Figures 2B–I, 3B–I, yellow arrows). Therefore, we finally diagnosed the patient with DS and AIS.

## Discussion

Diabetic striatopathy (DS) is a rare complication of diabetes mellitus (DM), characterized by a prominently increased blood glucose level, unilateral striatal hyper-density on CT, and hyperintensity on T1-weighted MRI, as well as contralateral choreiform movement (Dong and Zhang, 2021). Bilateral striatal lesions were rarely (9.7%) seen in patients with DS, which help distinguish DS from metabolic disorders, infectious, drugs, or toxics-induced striatal lesions that prominently affect the bilateral striatum (Chua et al., 2020; Dong and Zhang, 2021). DS prominently occurs in Asian women, particularly these patients with newly diagnosed or poorly controlled diabetes. Although most patients with DS were negative for ketone bodies, some patients still suffered from episodes of ketotic hyperglycemia (Tan et al., 2014; Markowska et al., 2021).

### Imaging Features

Striatal hyper-density on CT images and hyperintensity on T1-weighted MRI images are the representative neuroimaging findings (Chua et al., 2020). However, in the present case, the striatal anomalies on the initial CT and MRI images were not exactly consistent, which may be attributed to the different sensitivity (Lin et al., 2001). MRI seemed to be more sensitive; furthermore, striatal hyperintensity on MRI images tended to last longer than that on CT images (Lin et al., 2001; Chua et al., 2020). The striatal hyperintensity on T1-weighted images may achieve its maximum value on an average of 3 months, and hyperintensity began to decrease thereafter (Chua et al., 2020). This finding explained why an increased hyperintensity on T1 images was observed on the follow-up MRI, although the clinical symptoms have been completely relieved. The delayed resolution of T1 hyperintensity indicated that MRI may be a good tool for evaluating the presence of DS (Lin et al., 2001; Kitagawa et al., 2017; Zheng et al., 2020).

In addition to representative T1-weighted and CT images, we also recorded the dynamic changes of T2, FLAIR, DWI, and ADC images of patients with DS. In the early stages of onset, there was hypointensity on T2, FLAIR, ADC images, and equal signal intensity on DWI images (Figures 1D,E,G,H,M,N,J,K, red arrows), which were gradually increased and presented with slight hyperintensity on T2 and FLAIR images (Figures 2D,G, red arrows). Initial ADC hypointensity changed to equal signal intensity (Figure 2M, red arrows). Abnormal striatal signals finally disappeared (Figures 3A,D,G,J). Noticeably, the normalization of abnormal ADC signals was accompanied by a significant improvement in clinical symptoms, which was coherent with previous reports (Chu et al., 2002; Zheng et al., 2020). ADC sequence has been thought to be a tool to predict the prognosis of patients with DS (Chu et al., 2002; Zaitout, 2012).

Although the imaging presentations of DS are generally reversible in most patients, irreversible striatum lesions, such as caudate atrophy, have been occasionally reported in previous reports (Lucassen et al., 2017; Chatterjee et al., 2022). Long-term uncontrolled hyperglycemia is a major cause of irreversible striatal damage (Lucassen et al., 2017; Chatterjee et al., 2022). In this case, the imaging findings of the patient were only partially reversible. Caudate atrophy might present as a severe endpoint of DS, but it has rarely been reported in previous studies, possibly due to timely correction of hyperglycemia or lack of long-term follow-up in most cases (Lucassen et al., 2017).

Lacunar infarction in the striatum might be found in brain specimens from patients with DS, whereas imaging abnormalities indicative of lacunar infarction are often absent (Ohara et al., 2001; Abe et al., 2009). Imaging manifestations of DS and AIS occurring simultaneously in the striatum are easily misdiagnosed as AIS with hemorrhage, leading to delayed diagnosis and treatment. Dynamic imaging changes and detailed clinical data will help us distinguish DS from AIS with hemorrhage. However, it is still an enigma why a systemic metabolic disturbance leads to a unilateral symptom and why the striatum is particularly vulnerable to hyperglycemia.

### Pathophysiological Mechanism

Although the pathophysiological mechanism is still unclear, several hypotheses have been proposed, including microangiopathic lesions, metabolic disorders, autoimmune inflammatory response, neurodegeneration, dopamine, and estrogen involvement (Chang et al., 2010; Kumar Vadi et al., 2020; Zheng et al., 2020). The microangiopathic lesion hypothesis has been widely known and microvascular hemorrhage seems to play a critical role in the pathogenesis of DS (Abe et al., 2009; Chua et al., 2020). On the one hand, hyper-density on CT and hyperintensity on MRI strongly indicate the presence of hemorrhage and methemoglobin (Altafullah et al., 1990; Rindler et al., 2020). On the other hand, microhemorrhages are the most common pathological findings (Chua et al., 2020), featured by the presence of macrophages containing hemosiderin granules (Ohara et al., 2001), focal microhemorrhage (Nath et al., 2006; Mestre et al., 2007), hemosiderin deposition, and red blood cell extravasation (Nath et al., 2006; Mestre et al., 2007). More recently, the abnormal signal on susceptibility-weighted imaging was observed in the DS-associated striatum lesions, confirming the view of microhemorrhages (Tencer and Yum, 2021).

Recent lacunar infarctions with reactive astrocytosis were also frequent in the damaged striatum at autopsy (Ohara et al., 2001; Abe et al., 2009), which may be closely related to the hyaline degeneration, stenosis, and occlusion of the lenticulostriate arteries (Lin et al., 2001; Nath et al., 2006). In our case, chronic microvascular lesions in the striatum may be the common pathological mechanism of AIS and DS. Besides, the acute ischemic attack may be induced by hyperglycemia based on vascular stenosis by a series of pathophysiological mechanisms, including increased plasma osmolality and hyperviscosity, and also damaged the blood-cerebrospinal fluid barrier and impairment of vascular fibrinolysis (Hafez et al., 2014; Pasquel et al., 2020; Jiang et al., 2021). These processes may aggravate the DS-associated striatum damage at the same time, leading to progressive involuntary movement. Furthermore, it is reasonable to postulate that the occurrence of acute cerebral infarction exacerbates striatum damage, and eventually leads to atrophy of the left caudate nucleus. The presence of caudate atrophy and infarction focuses provides more evidence for the microangiopathic lesion hypothesis.

### Treatment

Hypoglycemic therapies are essential for symptom control; however, most patients need additional anti-chorea medications, including haloperidol, clonazepam, risperidone, trihexyphenidyl, and tetrabenazine (Abe et al., 2009; Chua et al., 2020; Zheng et al., 2020). When DS and AIS occur simultaneously, antiplatelet therapy or reperfusion therapy may be beneficial, but the safety needs to be fully assessed due to the presence of microvascular hemorrhage (Chua et al., 2020).

### Prognosis

Patients with DS usually have a good prognosis with complete amelioration after correction of the hyperglycemia. A few patients developed persistent chorea even under a backdrop of well-controlled diabetes mellitus or complete resolution of abnormal striatum signals, suggesting the presence of irreversible damage (Ahlskog et al., 2001; Wu et al., 2014; Roy et al., 2016; Lucassen et al., 2017; Chatterjee et al., 2022). Severe or uncontrolled hyperglycemia is associated with chorea relapse and the attacks of acute cerebrovascular events, such as acute cerebral infarction and hemorrhage, leading to a poor prognosis (Carrion and Carrion, 2013; Lucassen et al., 2017; Chua et al., 2020; Dong and Zhang, 2021). Carrion et. al reported a patient who suffered from a large cerebral infarction 2 weeks after DS onset due to non-compliance with diabetic therapy (Carrion and Carrion, 2013), suggesting active hypoglycemic therapy can effectively avoid disease progression.

## Conclusion

Patients with diabetes who present with the choreiform movements should consider the diagnosis of DS, especially in those with poor blood sugar control. DS and AIS could occur simultaneously after hyperglycemia, which requires clinicians to pay more attention to avoid misdiagnosis and delayed treatment.

## Data Availability Statement

The original contributions presented in the study are included in the article/Supplementary Material, further inquiries can be directed to the corresponding author.

## Ethics Statement

The studies involving human participants were reviewed and approved by the Medical Ethics Committee of the Second Affiliated Hospital of Henan University of Science and Technology. The patients/participants provided their written informed consent to participate in this study. Written informed consent was obtained from the individual(s) for the publication of any potentially identifiable images or data included in this article.

## Author Contributions

MY and XH contributed to the conception and design of the study and wrote the first draft of the manuscript. JQ, YL, and JL contributed to the acquisition and analysis of data. All authors contributed to manuscript revision, read, and approved the submitted version.

## Funding

This work was supported by the Medical Science and Technology Project of Henan Province (Grant Number: LHGJ20191241).

## Conflict of Interest

The authors declare that the research was conducted in the absence of any commercial or financial relationships that could be construed as a potential conflict of interest.

## Publisher's Note

All claims expressed in this article are solely those of the authors and do not necessarily represent those of their affiliated organizations, or those of the publisher, the editors and the reviewers. Any product that may be evaluated in this article, or claim that may be made by its manufacturer, is not guaranteed or endorsed by the publisher.
